# HER2-positive feline mammary carcinoma

**DOI:** 10.18632/aging.101015

**Published:** 2016-08-11

**Authors:** Cláudia Marques, Jorge Correia, Fernando Ferreira

**Affiliations:** Center for Interdisciplinary Research in Animal Health, Faculty of Veterinary Medicine, University of Lisbon, 1300-477 Lisbon, Portugal

**Keywords:** feline mammary carcinoma, HER2-positive subtype, sHER2, cancer model

Laboratory models have been crucial to increase knowledge about oncogenic mechanisms but due to the limitations of rodents and cell culture approaches, they are viewed as artificial models, whereas the spontaneous tumors in pets are empirically considered to be a more powerful and reliable tool. With the publication and full annotation of cat's genome [1], researchers showed that the genome of the cat was subjected to a very small number of genomic rearrangements when compared to the human genome, reinforcing the general accepted idea that feline mammary carcinomas (FMC) may be a good translational model, also based on their epidemiological and histopathological features that closely resemble those found in the more aggressive breast cancer subtypes.

Nowadays, the incidence of feline mammary carcinoma (FMC) is increasing, probably because of the humanized lifestyle of the cat, unregulated environmental factors and better prevention/efficient treatment of infectious diseases. Despite this scenario, few studies have evaluated the protein expression in FMC, in order to uncover new prognostic factors and discover possible therapeutic targets. In two recent studies, we identified in a pool of FMC, the six molecular subtypes described in breast cancer patients, using the broadly accepted IHC-based classification established by St. Gallen International Expert Consensus Panel [2,3]. The results demonstrated that cats with Luminal A mammary carcinomas showed less aggressive clinicopathological features, contrasting with the cats that had triple negative mammary carcinomas which were associated with the lowest survival and disease free survival times, as reported for humans. Additional data on disease progression revealed that lungs, pleura and liver are the organs mostly affected by metastases, with a large heterogeneity in protein expression being found in primary FMC and paired metastases, particularly in distant metastases, where both estrogen and progesterone receptors are usually downregulated, in contrast, to cytokeratin 5/6 and Ki-67 expression, leading to an enrichment of triple negative/basal-like lesions over luminal A/B metastases, similar to what is reported in woman.

Concerning the role of the feline homologue of human epidermal growth factor receptor-2 (HER2) in mammary carcinoma, some reports have brought fragmentary data revealing that the molecular mechanisms underlying HER2 tumorigenesis may not be fully conserved between cat and man. The overexpression of this transmembrane glycoprotein that belongs to the epidermal growth factor receptor family, is correlated with a rapid tumor growth and progression in breast patients and because of this, is routinely measured by immunohistochemistry and by in situ hybridization, since in the majority of the cases (80%), HER2 overexpression is associated with gene amplification. Published data reveal that 20-30% of human breast cancer patients display HER2 over-expression, prompting a therapy based on humanized monoclonal antibodies and tyrosine kinase inhibitors which improves survival rates. In cats, although the majority of HER2-positive mammary carcinomas are classified as either tubulo-papillary or solid adenocarcinomas, as human HER2-positive breast tumors and the incidence of HER2 overexpression in mammary carcinomas (20-40%) seems to be similar to the one that occurs in breast patients [3,4,5,6], being associated with the more aggressive clinical phenotypes, reports showed low HER2 mRNA levels and no gene amplification in HER2-positive FMC [4,5], suggesting that this subtype of tumors may be a suitable cancer model to study human HER2-positive breast carcinomas without gene amplification. Nevertheless, in our opinion, these results need to be confirmed by further research by a large number of samples, using improved IHC and FISH protocols and other molecular techniques as PCR/qRT-PCR.

Recently, five sequence variations in the HER2 gene region that encodes for the extracellular domain, comprising exons 17 to 20, were described with 2 non-redundant sequence variations being associated with malignant/metastatic-status specificity [7], raising the hypothesis of their utility as biomarkers in cancer diagnosis/prognosis and probably justifying future cases of immunotherapy resistance, as described in woman. In addition, haplotyping studies have also revealed loss of heterozygosity in malignant/metastatic tumor samples as seen in women, reinforcing the idea of using spontaneous FMC as a high fidelity model to study the biology of human breast cancer [7]. Finally, last February, we reported that in cats, a portion of the HER2 receptor is released into the bloodstream (sHER2) and that animals with HER2-positive mammary carcinomas show higher serum HER2 levels than cats having mammary carcinomas with a normal HER2 expression and healthy ones [6]. Interestingly, the optimal threshold value determined to discriminate cats with HER2-positive mammary carcinomas was 10 ng/ml, the same cutoff recommended for postoperative follow-up of patients with HER2-positive breast tumors.

In summary, the published data clearly show that more research on HER2-positive FMC is needed in order to fully prove that this pathology is a suitable cancer model. From the veterinary point of view, the high prevalence of HER2 overexpression in FMC can be a great opportunity to study specific target therapies directed against the HER2 receptor and to improve diagnostic tools based on tissue and serum HER2 status.

## References

[1] Davis B (2009). Genomics.

[2] Soares M (2016). Breast.

[3] Soares M (2016). Tumour Biol.

[4] Ordás J. (2007). BMC Cancer.

[5] Soares M (2013). Microsc Microanal.

[6] Soares M (2016). Oncotarget.

[7] Santos S (2013). PLoS One.

